# Comparison between Electrocardiographic and Earlobe Pulse Photoplethysmographic Detection for Evaluating Heart Rate Variability in Healthy Subjects in Short- and Long-Term Recordings

**DOI:** 10.3390/s18030844

**Published:** 2018-03-13

**Authors:** Basilio Vescio, Maria Salsone, Antonio Gambardella, Aldo Quattrone

**Affiliations:** 1 Biotecnomed S.C.aR.L., 88100 Catanzaro, Italy; basilio.vescio@biotecnomed.it; 2 Neuroimaging Unit, Institute of Molecular Bioimaging and Physiology of the National Research Council (IBFM-CNR), 88100 Catanzaro, Italy; salsonemaria@gmail.com; 3 Department of Medical and Surgical Sciences, Magna Græcia University, 88100 Catanzaro, Italy; a.gambardella@unicz.it

**Keywords:** electrocardiography, heart rate variability, photoplethysmography, pulse-to-pulse intervals

## Abstract

Heart rate variability (HRV) is commonly used to assess autonomic functions and responses to environmental stimuli. It is usually derived from electrocardiographic signals; however, in the last few years, photoplethysmography has been successfully used to evaluate beat-to-beat time intervals and to assess changes in the human heart rate under several conditions. The present work describes a simple design of a photoplethysmograph, using a wearable earlobe sensor. Beat-to-beat time intervals were evaluated as the time between subsequent pulses, thus generating a signal representative of heart rate variability, which was compared to RR intervals from classic electrocardiography. Twenty-minute pulse photoplethysmography and ECG recordings were taken simultaneously from 10 healthy individuals. Ten additional subjects were recorded for 24 h. Comparisons were made of raw signals and on time-domain and frequency-domain HRV parameters. There were small differences between the inter-beat intervals evaluated with the two techniques. The current findings suggest that our wearable earlobe pulse photoplethysmograph may be suitable for short and long-term home measuring and monitoring of HRV parameters.

## 1. Introduction

Heart rate variability (HRV) has been increasingly used over the last four decades as a biomarker for the assessment of autonomic functions and cardiac health. Commonly accepted guidelines [1] have been established in order to standardize methods for acquiring and processing HRV data. Electrocardiography (ECG) is the preferred technology used to derive heart rate and HRV information. Interbeat intervals (IBI) are measured as the time intervals between any two adjacent R peaks in ECG waves. They are thus also referred to as RR intervals. From RR sequences, normal sinus to normal sinus (NN) interbeat intervals are then extracted and NN sequences are analyzed in order to extract time-domain and frequency-domain measurements that help quantify cardiac autonomic regulation. 

ECG is generally acquired using disposable adhesive electrodes placed on the torso. In long-term acquisitions, during the wearing subject’s normal activity, ECG signals may be affected by artifacts, due to movement, contact or sweat. People may feel uncomfortable and show a very low compliance with electrodes stuck to their skin. The conductive gel applied to the electrodes may cause allergic reactions and affect time-dependent measurements due to drying. Moreover, the need for standard placement of electrodes requires a competent operator, as the incorrect positioning of an electrode may result in a faulty recording.

To overcome some of these problems and to facilitate the diffusion of portable and easily wearable devices, photoplethysmography (PPG) has been introduced as an alternative technique for measuring HRV through the surrogate measurement of pulse rate variability (PRV). PPG sensors are commonly worn on the fingers due to the high signal amplitude that can be achieved in comparison with other sites. However, this configuration is not well suited to continuous monitoring, as most daily activities involve the use of the fingers. 

The use of miniaturization technologies and the placement of a PPG sensor system entirely on the ear can help to prevent motion artifacts due to torsion or contact. PPG sensors and devices are non-invasive, as they have no electrical interaction with the human body. They are also cheaper and require less maintenance than ECG devices [2].

A typical PPG wave has peaks and valleys representative of blood volume changes in a peripheral tissue (finger or earlobe) due to blood pulses. PPG pulses can therefore be associated with ECG R peaks, although they are delayed because of transit time through blood vessels, and pulse-to-pulse (PP) intervals can be used as a reliable replacement for RR intervals in many applications [3].

Several ECG to PPG comparison studies have been carried out in order to assess the use of PPG-HRV as a valid replacement, within certain limits, for ECG-HRV. Previous studies have investigated the comparison between short-term ECG- and PPG-HRV recordings. In a study by Jeyhani et al. [4], HRV analysis was applied to beat-to-beat intervals obtained from ECG and finger PPG from 19 healthy subjects. In Jeyhani’s study, PP intervals were computed on both the PPG signal and its second derivative. HRV measures derived from PP data showed little relative errors compared to those obtained from ECG NN intervals, except for pNN50, which showed a relative error of about 30%. Other authors [5] performed a similar comparison on two healthy subjects, with repeated measurements from a dedicated PDA device, capable of simultaneous ECG and PPG acquisition. A rigorous comparative analysis was carried out in another work [6] on 5-min sequences acquired from 10 healthy people at rest. No study, however, has focused on a comparison between HRV and ECG in long-term monitoring applications. 

In the present work, classical NN intervals derived from ECG are compared to PP intervals derived from a new PPG device that converts PPG waves into electrical pulses. The use of such a simplified electronic circuit enables very small wearable devices to be developed, without the need for ADC conversion depth and computational power for peak extraction from PPG waveforms. We also compared ECG-HRV and PPG-HRV both in short-term (up to 20 min) and long-term (24-h) recordings.

## 2. Materials and Methods

**Figure 1:**
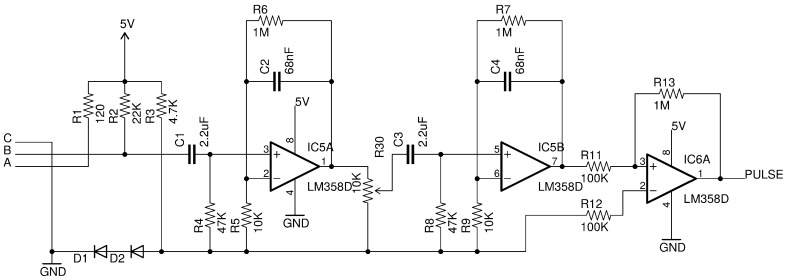
Analog circuit for filtering and amplification of the photoplethysmography (PPG) signal and for the generation of heartbeat pulses.

Simultaneous ECG and PPG recordings were taken from two different groups of people, both made up of 10 healthy subjects (five males + five females), without arrhythmias, aged from 25 to 62 years. The first group was monitored for 20 min at rest. The second group was monitored for 24 h during normal daily activity. ECG signals were recorded using an eMotion Faros 180° (Mega Electronics Ltd., Kuopio, Finland), set up to acquire data at a 1 KHz rate. This device has an internal QRS annotator that marks RR (NN) intervals. PPG data were recorded using a custom battery-operated device, based on an Arduino microcontroller and an analog circuit (Figure 1).

**Figure 2:**
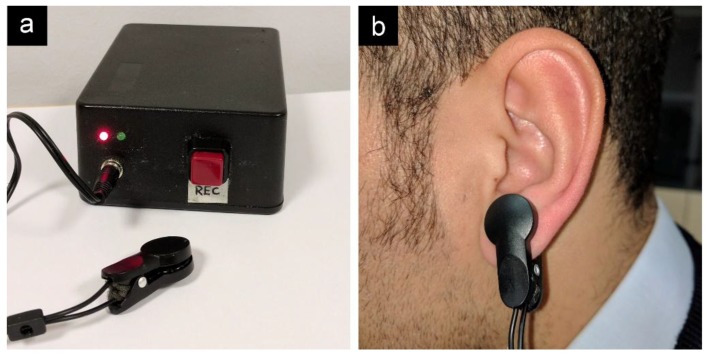
(**a**) Experimental device used for PPG acquisition, with Kyto HRM-2511B sensor clip, computation and storage of pulse-to-pulse (PP) intervals; (**b**) PPG sensor worn on left earlobe.

The PPG sensor was a Kyto HRM-2511B (Figure 2a), made up of an IR LED (LL-AR180IRC-2A) and a phototransistor (LL-AR180PTC-1A) mounted on the opposite sides of a clip that is worn on the earlobe (Figure 2b) and wired to the microcontroller box.

During the 24-h acquisition, the device was carried in a crossbody purse and the sensor was fixed to the earlobe by a piece of adhesive plaster. Another piece of plaster was used to fix the sensor wire near or on the neck in order to avoid motion artifacts. The whole system weighed less than 100 g.

The analog circuit performs the following functions: biasing of the LED connected to terminal A, biasing of the phototransistor connected to terminal B, filtering and amplification of the signal, and pulse conversion through the comparator stage. The signal from the phototransistor is filtered and amplified through a two-stage active bandpass filter. When the signal at the non-inverting input of the comparator exceeds the threshold of the two diodes (typically 1.2 V), a pulse is generated. The output of the comparator is connected to an interrupt line of the microcontroller. When a rising edge is detected, an interrupt service routine is fired, calculating the milliseconds elapsing from the previous pulse. This method does not entail sampling an analog waveform, thus enabling PP data to be generated directly from the analog circuit, while the microcontroller only records the times at which pulses are presented at one of its interrupt lines. Data are recorded on an SD card. The assembled device is shown in Figure 2a. 

For each subject in the first group, RR (NN) and PP data were simultaneously recorded for 20 min, at rest. In the second group, simultaneous 24-h recordings were taken continuously during normal daily activity (work, home, sleep, etc.). Sequences of interbeat intervals derived from ECG and PPG were aligned manually.

RR sequences that are less than 15 min in length are generally referred to as short-term data [7]. Five minutes is the minimum requirement for a reliable estimation of low frequency (LF) power [8]. Filtering out motion artifacts may result in shorter sequences for the analysis. In order to obtain sufficient information, 10-min segments were extracted from each recording, with a 0% to 50% overlap between consecutive segments. All 20-min acquisitions were divided into three segments (overlap = 50%), while a 0% overlap was used for 24 h acquisitions, resulting in about 144 segments for each subject. For each segment, the following time and frequency HRV domain measures were calculated:

- AVNN: average of all NN intervals;
- SDNN: standard deviation of all NN intervals;
- rMSSD: square root of the mean of the squares of differences between adjacent NN intervals;
- pNN50: percentage differences between adjacent NN intervals greater than 50 ms;
- Low frequency (LF) power: the spectral power of all NN intervals between 0.04 and 0.15 Hz;
- High frequency (HF) power: the spectral power of all NN intervals between 0.15 and 0.4 Hz;
- LF/HF ratio: the ratio of low to high frequency power;
- SD1, SD2: dispersions (standard deviations) of points along orthogonal axes of a fitted ellipse on Poincaré maps. Poincaré plots are one of the most common techniques in nonlinear HRV analysis [6]. In a Poincaré diagram, each RR interval is plotted against the previous interval. Good quality RR signals produce plots with all data points clustered together. Corrupted RR signals will result in Poincaré maps with scattered data points [5].

RR data from segments in short-term acquisitions were processed using PhysioNet HRV Toolkit routines. Artifact removal by HRV Toolkit is described in [7]. First, RR intervals that are less than 0.4 s or more than 2.0 s are excluded. Next, using a window of 41 intervals (20 intervals on either side of the central point), the average over the window is calculated excluding the central interval. If the central interval lies outside 20% (0.2) of the window average, this interval is flagged as an outlier and excluded. These parameters (minimum and maximum RR duration, window length and percentage threshold) can be customized by the user. Defaults are used. Artifacts in 24-h acquisitions were removed by means of Kubios HRV [9,10] thresholding algorithms, as described in [9,10]. The threshold-based artifact correction algorithm in Kubios HRV compares each RR interval value against a local average interval. The local average is obtained by median filtering the RR interval time series, and the local average is therefore not affected by single outliers in the RR interval time series. If an RR interval differs from the local average by more than a specified threshold value, the interval is identified as an artifact and is marked for correction. The threshold value (expressed in seconds) can be selected from very low: 0.45 s; low: 0.35 s; medium: 0.25 s; strong: 0.15 s; very strong: 0.05 s; and custom, for setting a custom threshold in seconds. The correction is made by replacing the identified artifacts with interpolated values using a cubic spline interpolation. By default, the software applies no correction. The threshold is selected individually, based on the average heart rate and presence of artifacts, as suggested in the manual. The low-to-medium correction was used. After correction with Kubios HRV, each extracted segment was corrected again using the HRV Toolkit before computing HRV measures. About 1% to 5% of data were excluded after this second stage correction for each of the subjects involved in the 24-h recordings.

Calculations of HRV measures on all corrected segments were performed using the PhysioNet HRV Toolkit [7,11], and power spectra were computed by means of the Lomb–Scargle periodogram for unevenly sampled data.

In order to compare HRV measures within subjects in short term acquisitions, the one sample *t*-test and the Wilcoxon signed rank test were used for all of the three overlapping segments, after checking distribution properties by means of the Shapiro–Wilk test. 

HRV measures on 24-h acquisitions from all subjects in the second group were concatenated (thus resulting in sequences of about 1440 measurements) and compared by means of Pearson’s correlation tests. Agreement coefficients were evaluated on Bland-Altman statistics according to [12]. Linear regression was used to assess differences in slope from 1. 

The significance level was set at α = 0.05. Comparisons between curves were performed using Octave (GNU Octave for Linux, version 3.8.2, The Free Software Foundation, Boston, MA, USA, 2014). Statistical analysis was carried out using R (R for Unix/Linux, version 3.1.1, the R Foundation for Statistical Computing, Vienna, Austria, 2014). Original acquisitions are provided as Supplementary Materials. All subjects from whom signals were acquired gave their informed consent for inclusion before they participated in the study. The study was conducted in accordance with the Declaration of Helsinki, and the protocol was approved by the Regional Ethics Committee of the Region of Calabria (No. 115, 27 April 2017).

## 3. Results

In short-term acquisitions (made up of three segments, each lasting 10 min), each PP curve was compared to its related NN curve. Cross-correlation coefficients, together with root mean square errors (RMSE), were computed for each subject and for each segment, and the means and standard deviations on the sample of 10 subjects are shown in Table 1.

**Figure 3:**
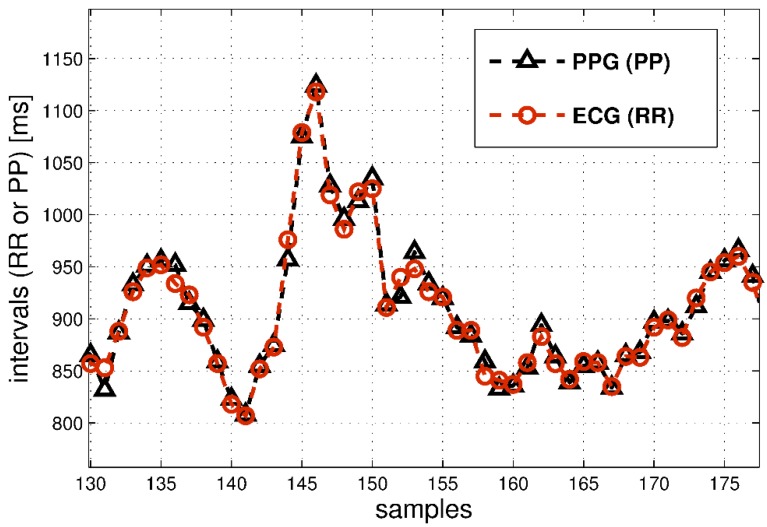
Example signals (about 40 s) acquired with PPG device and reference ECG device for the same subject.

These results showed a superposition between each PP-RR couple of curves. An example of such comparison is reported in Figure 3, for an acquisition sample of about 40 s.

As reported in a previous study [4], errors in PPG HRV measures relative to ECG HRV measures were evaluated, and average percentage errors in the 10-subject sample are reported in Table 2. 

The largest errors were found in pNN50, in segments 2 and 3. In all three segments, the percentage error on pNN50 also showed the largest dispersion.

**Figure 4:**
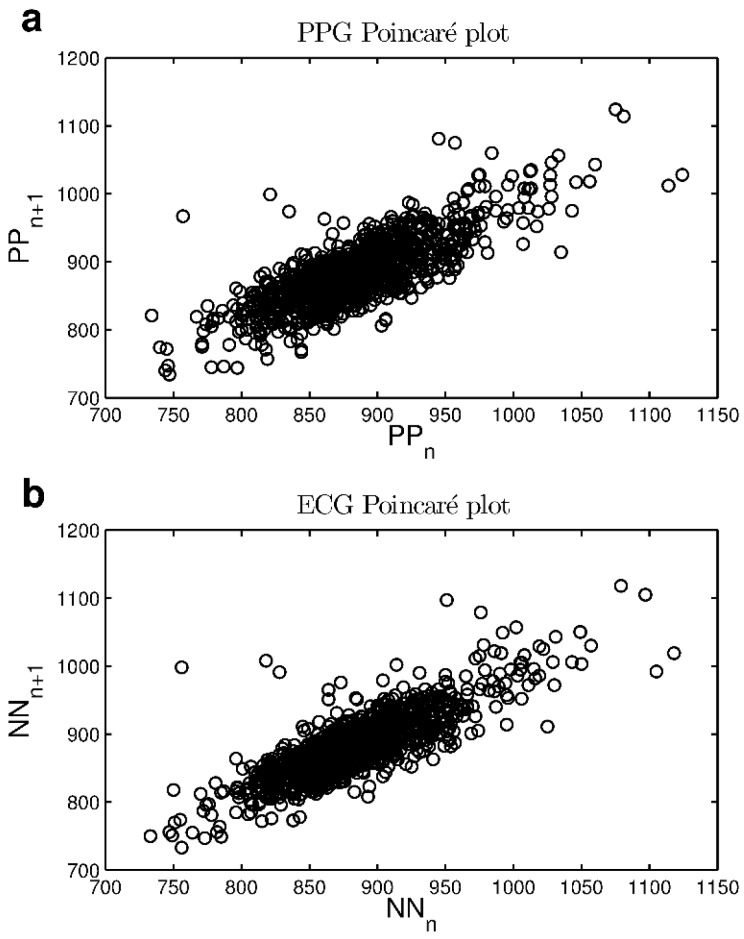
Example of Poincaré plots of 20 min recordings, for the same subject: (**a**) PPG PP intervals and (**b**) ECG NN intervals.

The Poincaré plots also appear to be comparable, as highlighted in Figure 4, where PPG-derived (Figure 4a) and ECG-derived (Figure 4b) maps are shown for an example subject. Points are well-clustered and ellipse-shaped for both signals.

A more significant comparison between ECG and PPG time-domain and frequency-domain HRV measures is shown in Table 3. 

Paired tests highlighted that there were no significant differences between PPG-based and ECG-based HRV parameters in all three segments, except for AVNN (*p* = 0.01, *p* < 0.001 and *p* < 0.001) and LF/HF ratio (*p* = 0.002, *p* = 0.002 and *p* = 0.02). In these cases, a low *p* value is determined by the high sensitivity of the paired test to small shifts in the median. Table 4 shows correlations with r > 0.92 between PPG-based HRV and ECG-based parameters, also for AVNN.

**Figure 5:**
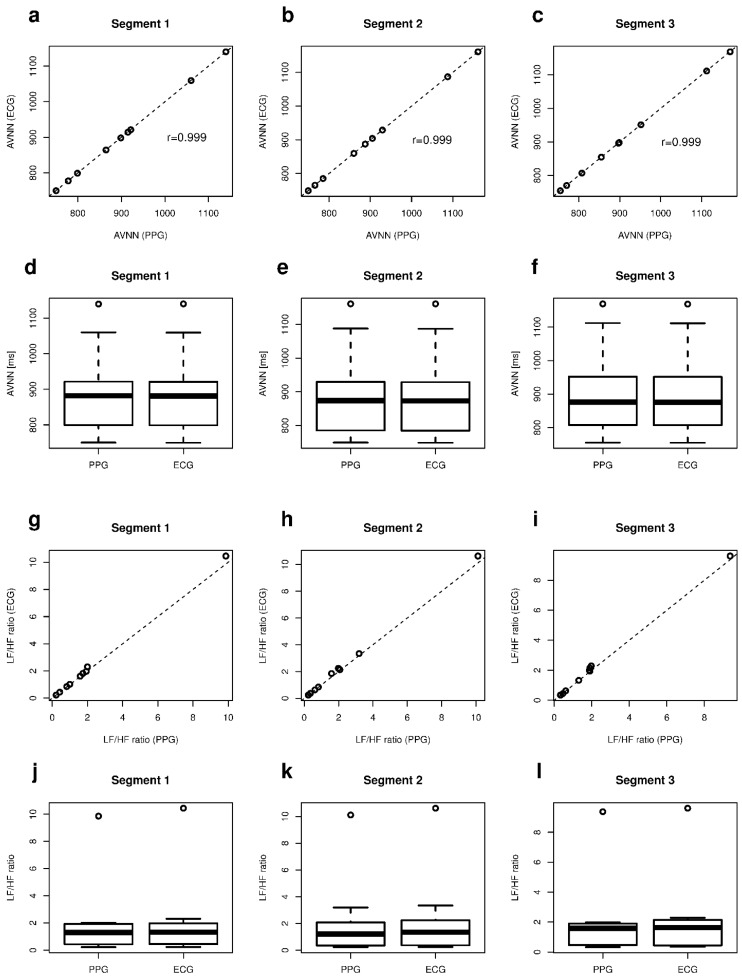
Short-term acquisitions: (**a**–**c**) Correlation of PPG AVNN with ECG AVNN; (**d**–**f**) Boxplots of PPG AVNN and ECG AVNN; (**g**–**i**) Correlation of PPG LF/HF ratio with ECG LF/HF ratio; (**j**–**l**) Boxplots of PPG LF/HF ratio and ECG LF/HF ratio.

The correlation between AVNN values in all three segments is also shown in Figure 5a–c. In Figure 5d–f boxplots are drawn, highlighting the matching between PPG and ECG AVNN data. 

**Figure 6:**
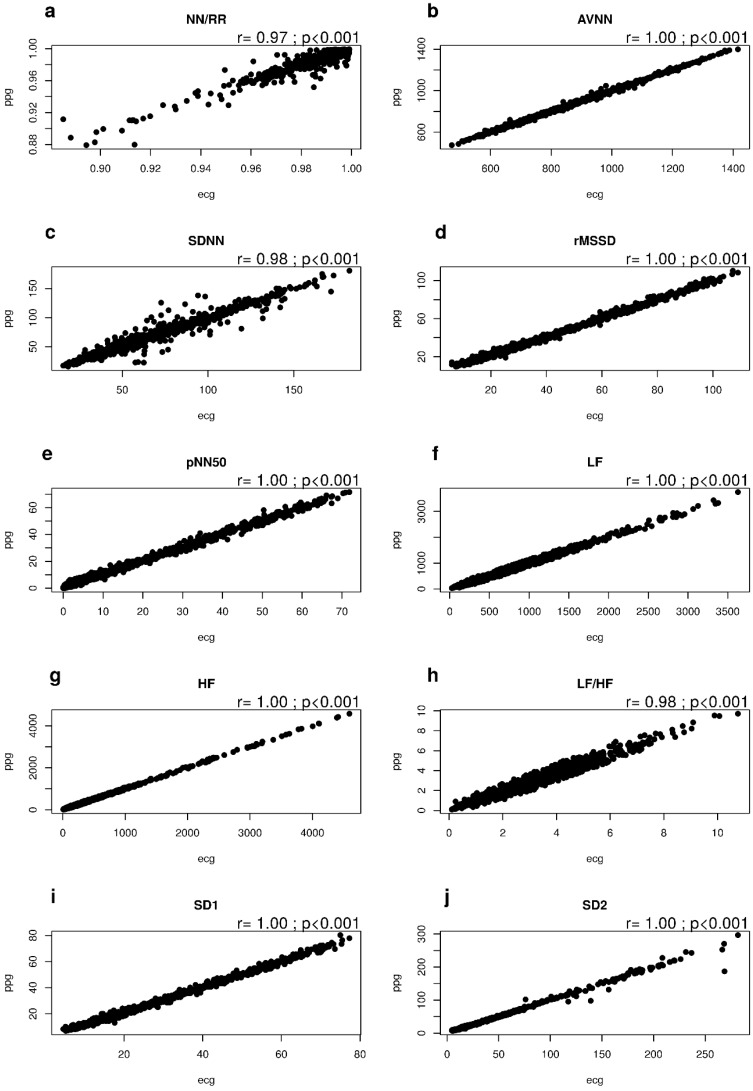
Correlations between ECG and PPG HRV measurements from 24-h acquisitions from 10 healthy subjects: (**a**) NN/RR ratio; (**b**) AVNN; (**c**) SDNN; (**d**) rMSSD; (**e**) pNN50; (**f**) LF power; (**g**) HF power; (**h**) LF/HF ratio; (**i**) SD1; (**j**) SD2.

Long-term acquisitions (24-h recordings) were processed after extracting all adjacent 10-min segments (overlap = 0%). For each of the ten subjects, and for each of the HRV measures considered, two sequences were computed, one for the ECG and one for the PPG data. Correlations were found between each couple of PPG-based and ECG-based sequences of HRV measures with r ≥ 0.97, as shown in Figure 6. 

**Figure 7:**
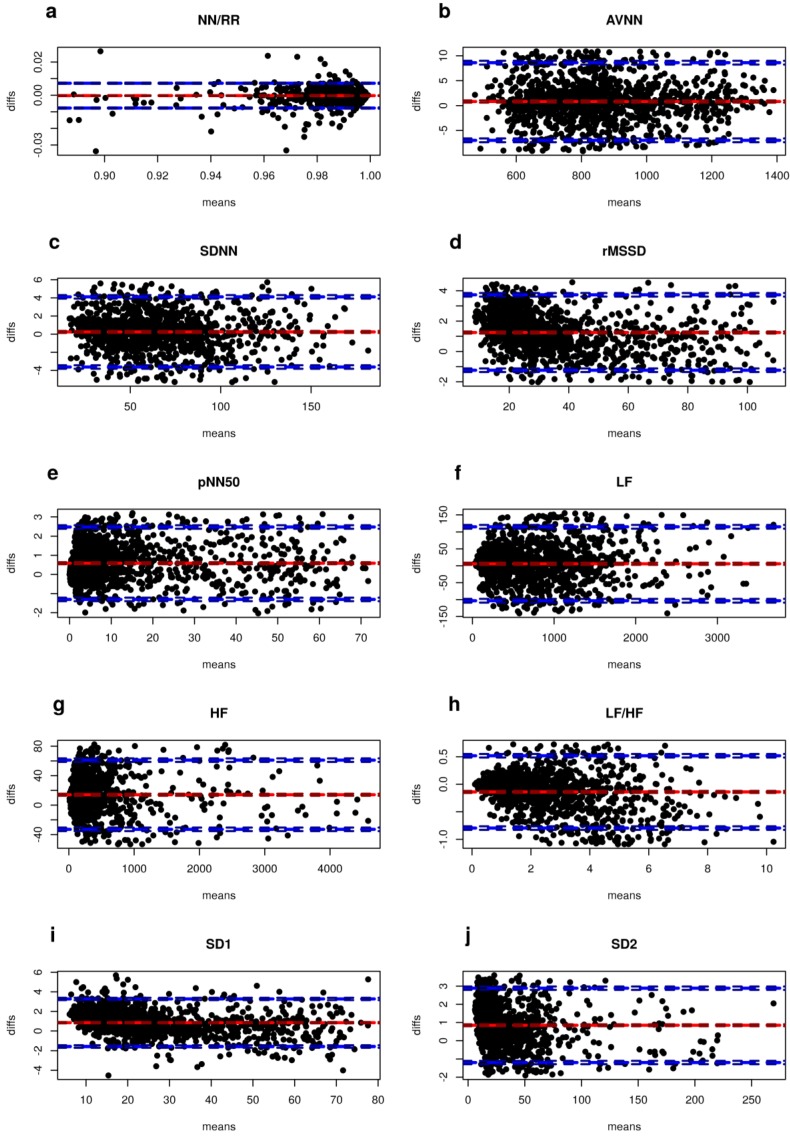
Bland–Altman plots comparing ECG and PPG HRV measurements from 24-h acquisitions in 10 healthy subjects: (**a**) NN/RR ratio; (**b**) AVNN; (**c**) SDNN; (**d**) rMSSD; (**e**) pNN50; (**f**) LF power; (**g**) HF power; (**h**) LF/HF ratio; (**i**) SD1; (**j**) SD2.

Figure 7 shows Bland–Altman plots for 24-h data (all 10 subjects). The percentage number of points that were found to lie beyond ±1.96 standard deviations from the mean difference were 4.5% for NN/RR ratio, 8.7% for AVNN, 7.4% for SDNN, 6.0% for rMSSD, 7.5% for pNN50, 7.7% for LF power, 7.4% for HF power, 8.2% for LF/HF ratio, 6.2% for SD1 and 6.7% for SD2.

Table 5 reports the average RMSE, slope test and coefficient of agreement for all variables, acquired from all ten subjects involved in 24-h acquisitions. ECG measures are used as the reference.

Slopes comparable with unity were found for the NN/RR ratio, AVNN, LF power and HF power (*p* > 0.05). A coefficient of agreement was computed as the ratio of half the range of the limits of agreement and the mean of the pairwise measurement means evaluated from Bland-Altman statistics in all the ten subjects, as suggested in [12]. A good (coefficient ≤ 0.1) to acceptable (0.1 < coefficient ≤ 0.2) agreement was found for all variables except the LF/HF ratio, according to the definitions given in [12].

## 4. Discussion

Other studies have shown evidence of PPG pulse rate variability as a valid surrogate of ECG-HRV [13,14,15,16,17,18,19,20]. Gil et al. [17], showed that this also holds in non-stationary conditions, at least during the tilt table test. However, other authors [21,22,23,24] have critically examined this technique, pointing out various limitations. For example, Constant et al. [22] observed a respiratory rhythm in PRV signals that has no equivalent in ECG HRV. They used a Finapres device for PPG pulse rate variability detection, however, which has a rather low sampling frequency. Another major limitation of the PPG is the uncertainty in the correct location of a fiducial point for the detection of complexes, mainly due to variations in peripheral arterial properties, as in the case of local cold exposure [25]. 

In the present work, a comparison of classical ECG-HRV with PPG-HRV was introduced, using a customized device that converts the PPG signal generated by a LED-photodiode couple placed on the earlobe of a healthy subject into electric pulses. In order to limit the presence of artifacts, the PPG-HRV sensor was fixed to the earlobe with a couple of adhesive patches. The analog conversion circuit filters and amplifies the signal, comparing it to a fixed threshold. When this reference level is exceeded, a pulse is generated at the output and an interrupt request is fired. This threshold is determined by a couple of 1N4148 diodes, thus resulting in about 1.2 V. On a 5 V logic device such as the Arduino platform, this turns out to be closer to the baseline level of the PPG waveform complex, rather than to its peak. This ensures a more stable fiducial point for the measurement of PP intervals, with a better response to arterial changes due, for example, to thermal variations [25]. This reference level is also not influenced by fluctuations in the baseline level, which could lead to false pulse detections. The interrupt driven evaluation of the time between subsequent pulses charges the microcontroller with very little computational load. This suggests that this logic is easily implementable in systems with fewer resources and far less power consumption. Modern system-on-chip (SOC) devices, such as the Cypress PSOC 4100, have the capacity to manage mixed signals, thanks to the integration of a programmable analog front-end (AFE) with operational amplifiers. Such systems also include a Cortex M0 microcontroller and a Bluetooth low energy module, thus making it possible to re-implement the whole system on a few mm^2^ surface and with connectivity to mobile devices. It can therefore easily be scaled to be entirely worn on an ear, with no need for wires and other parts, thus enhancing compliance and robustness to artifacts.

In the system implementation used for the present study, the total current needed to power all the electronics is about 50 mA, mostly required by the LED. Using a programmable system-on-chip (PSOC) and an optimized LED-photodiode sensor, the drawn current can be scaled down by a factor of 4 or 5, enabling a very small battery to be used and ensuring a very small size. It could thus be easily and entirely worn on the ear and the monitoring could be continuous for more than 24 h.

With the current implementation, it is also possible to perform long-term recordings and to monitor fluctuations in the autonomic activity over a relatively long time or an entire circadian cycle. In the present study, overlapping segments of NN and PP signals were analyzed. When considering frequency HRV parameters, a time–frequency analysis is performed, and the time resolution can be increased by overlapping segments, though the computational performance may be degraded. For the moment, long term parameters have been excluded from the comparison, as this study considers short term fluctuations in HRV measures over longer time intervals.

Curve comparisons and HRV parameter comparisons yielded comparable results with previous works [3,4,5,6]. The cross correlation coefficients and RMSE shown in Table 1 suggest that the acquired NN and PP series are very close to each other, as is also shown by the Poincaré maps (e.g., Figure 4). 

Percentage errors in Table 2, for each segment of interbeat intervals, also showed agreement between the two measuring systems, which was enforced by paired tests in Table 3. Errors were shown to be comparable to those found in [5], where a professional acquisition system was used for PPG, except for pNN50, whose percentage error was about one half that of our error. Smaller errors were found than in [4] on SD1, SD2, pNN50, SDNN and rMSSD, although pNN50 was confirmed as having the highest percentage error. Percentage errors on LF power, HF power and LF/HF ratio were also higher than errors on other measures, but paired tests revealed no significant differences, except for the LF/HF ratio. 

Only AVNN values seemed to be significantly different; however, this was mainly due to the sensitivity of paired tests to shifts in the mean (median) value of the difference between two observations from zero. In this case, the difference between AVNN values had a very low dispersion around its mean value, for each segment (segment 1, 0.48 ± 0.36 ms; segment 2, 0.49 ± 0.23 ms; segment 3, 0.56 ± 0.16 ms). Correlations, in fact, confirmed a significant match between measurements. This was also observed in long-term acquisitions from 24-h continuous monitoring (Table 5), where good-to-acceptable coefficients of agreement were determined from Bland–Altman statistics. 

However, a poor agreement was found for the LF/HF ratio. Bland–Altman plots (Figure 7) showed that more than 90% of points showed randomly distributed differences within ±1.96 standard deviations from the mean difference. Very little literature is available for comparisons between HRV measures from ECG and PPG sensors in long-term monitoring applications. 

Some studies have recently assessed the performances of wearable devices, such as smart bands, in monitoring physiological changes while walking, running or sleeping [26]. However, such devices have strong limitations, such as low perfusion in the wrist area and unstable beat detection due to motion [2]. Most studies focus on signal processing techniques for error compensation on the detected RR [27], however no one has yet assessed series of HRV measures from long-term PPG acquisition by comparing them to those derived from ECG recordings. 

The results shown in the present study suggest that our PPG-HRV measurements appear to be strictly comparable to ECG-HRV measurements. This does not mean, however, that the proposed technique should replace ECG-HRV in clinical settings and in medical devices, where electrocardiography is still the gold standard. However, given these limitations, our PPG-HRV approach could be used in continuous monitoring approaches for the evaluation of fluctuations in autonomic activity. It can also be easily implemented, miniaturized and configured as a wearable continuous monitoring system in several daily applications.

## 5. Conclusions

In this paper, we have presented a simple method for the evaluation of beat-to-beat time intervals and pulse rate variability, which was compared to a standard method for the evaluation of RR intervals and heart rate variability. We compared a portable, wearable ECG-HRV with a new customized wearable PPG-HRV device, both in short-term and long-term acquisition settings. 

To our knowledge, this is the first time that an ear-worn PPG-HRV device, capable of generating interbeat intervals through interrupts driven by analog pulses, has been compared to ECG, both in short- and in long-term monitoring. Correlations with r > 0.9 were found between ECG-HRV and PPG-HRV measures. In long-term recordings, a linear relationship with a slope comparable to unity and a coefficient of agreement ≤0.2 were also found. We assessed our customized pulse photoplethysmographic device as a potential surrogate for ECG in the evaluation of heart rate variability measures. Although it is not meant to be a replacement in clinical applications, it can be used in all those monitoring applications where several conditions need to be assessed over 24-h cycles, such as quality of life, stress load in working environments, night sleep quality, as well as for screening autonomic and cardiac dysfunctions. It can also be easily implemented in a miniaturized form and is wearable on the ear, which is a peripheral region with good vascularization and perfusion properties. It thus ensures a good-quality signal, robustness and the very low risk of errors and artifacts in both short-term and long-term monitoring applications.

## Figures and Tables

**Table 1:** Curve correlation coefficients and root mean square errors in short-term acquisitions.

	Segment 1	Segment 2	Segment 3
correlation coefficients	0.996 ± 0.003	0.996 ± 0.003	0.996 ± 0.005
RMSE [ms]	4.405 ± 1.877	4.173 ± 1.604	4.331 ± 1.670

**Table 2:** Percentage errors of heart rate variability (HRV) measurements in short-term acquisitions.

Error%	Segment 1	Segment 2	Segment 3
	Mean ± std.dev.	Mean ± std.dev.	Mean ± std.dev.
NN/RR ratio	0.10 ± 0.16	−0.01 ± 0.12	−0.03 ± 0.09
AVNN	0.06 ± 0.04	0.06 ± 0.02	0.06 ± 0.02
SDNN	0.23 ± 0.57	0.16 ± 0.55	0.19 ± 0.88
rMSSD	0.23 ± 3.00	0.41 ± 3.15	0.27 ± 2.81
pNN50	0.22 ± 9.98	6.05 ± 15.57	4.89 ± 12.85
LF power	−0.76 ± 2.83	−1.74 ± 2.74	−0.86 ± 2.16
HF power	3.37 ± 5.64	3.82 ± 4.68	3.84 ± 5.56
LF/HF ratio	−3.85 ± 3.55	−5.22 ± 4.25	−4.30 ± 5.21
SD1	−1.07 ± 2.58	0.44 ± 3.20	0.34 ± 2.72
SD2	−0.00 ± 0.41	0.06 ± 0.46	0.17 ± 0.39

NN: normal sinus to normal sinus; AVNN: average of all NN intervals; SDNN: standard deviation of all NN intervals; rMSSD: square root of the mean of the squares of differences between adjacent NN intervals; pNN50: percentage differences between adjacent NN intervals greater than 50 ms; LF: low frequency; HF: high frequency; SD: standard deviations.

**Table 3:** Comparison of HRV measurements from 20-min acquisitions.

	PPG, Mean ± std.dev.	Reference, Mean ± std.dev.	*p* Value
Segment 1			
NN/RR ratio	0.994 ± 0.007	0.993 ± 0.008	0.181 *
AVNN [ms]	899.144 ± 122.069	898.662 ± 122.258	0.011 *
SDNN [ms]	48.677 ± 19.09	48.576 ± 19.083	0.336 ^†^
rMSSD [ms]	37.753 ± 17.667	38.003 ± 18.768	0.569 ^†^
pNN50 [%]	17.44 ± 14.876	17.741 ± 15.345	0.29 ^†^
LF power [ms^2^]	654.396 ± 560.816	662.423 ± 576.355	0.378 ^†^
HF power [ms^2^]	746.437 ± 731.819	740.884 ± 745.707	0.381 ^†^
LF/HF ratio	1.984 ± 2.852	2.093 ± 3.028	0.002
SD1 [ms]	30.264 ± 16.947	30.89 ± 17.984	0.322 *
SD2 [ms]	65.722 ± 28.181	65.792 ± 28.435	0.495 ^†^
Segment 2			
NN/RR ratio	0.996 ± 0.003	0.996 ± 0.003	1 *
AVNN [ms]	899.109 ± 134.005	898.624 ± 134.072	<0.001 ^†^
SDNN [ms]	47.964 ± 16.901	47.921 ± 16.997	0.601 ^†^
rMSSD [ms]	37.201 ± 17.662	37.377 ± 18.721	0.685 ^†^
pNN50 [%]	16.345 ± 14.372	16.139 ± 14.766	0.546 ^†^
LF power [ms^2^]	659.338 ± 556.892	672.83 ± 564.52	0.064 *
HF power [ms^2^]	726.809 ± 811.377	717.345 ± 829.483	0.242 ^†^
LF/HF ratio	2.122 ± 2.974	2.252 ± 3.121	0.002 *
SD1 [ms]	28.939 ± 16.707	29.169 ± 17.744	0.77 *
SD2 [ms]	63.831 ± 23.963	63.851 ± 24.204	0.86 ^†^
Segment 3			
NN/RR ratio	0.996 ± 0.004	0.997 ± 0.003	1 *
AVNN [ms]	907.195 ± 137.581	906.637 ± 137.495	<0.001 ^†^
SDNN [ms]	52.471 ± 18.432	52.444 ± 18.698	0.863 ^†^
rMSSD [ms]	38.606 ± 16.339	38.737 ± 17.256	0.767 ^†^
pNN50 [%]	17.736 ± 13.687	17.371 ± 13.859	0.259 ^†^
LF power [ms^2^]	767.526 ± 583.003	774.654 ± 585.287	0.375 *
HF power [ms^2^]	734.273 ± 657.889	728.607 ± 704.71	0.084 *
LF/HF ratio	2.006 ± 2.682	2.106 ± 2.75	0.02 *
SD1 [ms]	30.004 ± 15.936	30.181 ± 16.885	0.635 ^†^
SD2 [ms]	70.733 ± 26.226	70.653 ± 26.352	0.443 ^†^

* Shapiro–Wilk test followed by Wilcoxon (paired) signed rank test. ^†^ Shapiro-Wilk test followed by one sample (paired) *t*-test.

**Table 4:** Short-term acquisitions, correlations of HRV measurements.

	Segment 1	Segment 2	Segment 3
	Coeff. (*p* Value)	Coeff. (*p* Value)	Coeff. (*p* Value)
NN/RR ratio	0.992 (<10^−6^)	0.928 (0.0001)	0.976 (10^−6^)
AVNN	0.999 (<10^−6^)	0.999 (<10^−6^)	0.999 (<10^−6^)
SDNN	0.999 (<10^−6^)	0.999 (<10^−6^)	0.999 (<10^−6^)
rMSSD	0.999 (<10^−6^)	0.999 (<10^−6^)	0.998 (<10^−6^)
pNN50	0.999 (<10^−6^)	0.998 (<10^−6^)	0.998 (<10^−6^)
LF power	0.999 (<10^−6^)	0.999 (<10^−6^)	0.999 (<10^−6^)
HF power	0.999 (<10^−6^)	0.999 (<10^−6^)	0.999 (<10^−6^)
LF/HF ratio	0.999 (<10^−6^)	0.999 (<10^−6^)	0.999 (<10^−6^)
SD1	0.999 (<10^−6^)	0.999 (<10^−6^)	0.999 (<10^−6^)
SD2	0.999 (<10^−6^)	0.999 (<10^−6^)	0.999 (<10^−6^)

**Table 5:** Errors, linearity and agreement of RR and HRV measurements from 24-h acquisitions.

No. = 10 Subjects	RMSE	Slope Test (H0: Slope = 1)	Coefficient of Agreement
	Mean ± std.	*p* Value	
RR values	5.80 ± 1.06	0.514	0.014
NN/RR ratio	0.004 ± 0.0005	0.088	0.008
AVNN	8.21 ± 0.53	0.243	0.019
SDNN	6.42 ± 0.46	<0.001	0.173
rMSSD	2.01 ± 0.13	<0.001	0.088
pNN50	1.46 ± 0.11	<0.001	0.174
LF power	120.33 ± 10.83	0.098	0.138
HF power	109.14 ± 21.31	0.085	0.111
LF/HF ratio	0.60 ± 0.17	<0.001	0.259
SD1	1.46 ± 0.08	<0.001	0.091
SD2	4.51 ± 2.88	<0.001	0.171

## Supplementary Materials

The following are available online at http://www.mdpi.com/1424-8220/18/3/844/s1. 20-min and 24-h acquisitions of both ECG and PPG signals are provided as Matlab/Octave workspace files or ASCII files in a single archive.
